# Analysis of play in children aged 2 to 4 with developmental language disorder

**DOI:** 10.1590/2317-1782/e20240098en

**Published:** 2026-04-10

**Authors:** Júlia França Viana, Tainá Rossato Benfica, Gustavo Marcelino Siquara, Ana Paula Ramos de Souza

**Affiliations:** 1 Departamento de Saúde e Comunicação Humana, Universidade Federal do Rio Grande do Sul – UFRGS - Porto Alegre (RS), Brasil.; 2 Programa de Pós-graduação em Distúrbios da Comunicação Humana, Universidade Federal de Santa Maria – UFSM - Santa Maria (RS), Brasil.; 3 Programa de Pós-graduação em Medicina e Saúde Humana, Escola Bahiana de Medicina – EBM - Salvador (BA), Brasil.

**Keywords:** Games and Toys, Child Development, Socioeconomic Factors, Developmental Language Disorder, Gestational Age

## Abstract

**Purpose:**

To analyze playing in children aged two to four years and eleven months with Developmental Language Disorder (DLD), and to investigate correlations between play, development dimensions, and the variables maternal education, family income and gestational age.

**Methods:**

This is quantitative, cross-sectional research, with 37 children aged between two and four years and eleven months, diagnosed by speech therapists with DLD. This diagnosis was confirmed using two instruments: the Labyrinth Scale which allows a differential assessment between DLD and Autism Spectrum Disorder (ASD) and the Dimensional Inventory of Child Development (IDADI) wich allows confirmation of language development delay, as well as assessment of other dimensions of child development. Play was assessed by analyzing the filming of the interaction between the child and the researcher during the collection of the Labyrinth Scale, considering expected levels for chronological age from two to five years. Based on the identified categories, descriptive and statistical analyses were performed on the variables maternal education, gestational age and family income.

**Results:**

Most children presented a delay of one level or more in play development, accompanied by a significant delay in expressive language. There was a statistical association between play and the fine motor dimension of child development.

**Conclusion:**

Children with DLD showed delay in play, and better development in play was positively associated with better performance in fine motor skills, suggesting its relevance as a space for cognitive development and subjectivation.

## INTRODUCTION

It is undeniable that play is widely recognized as fundamental to child development. Play is considered a universal practice that exhibits distinct characteristics within social, historical, and cultural contexts^(1,2)^. In this sense, play assumes a significant role in the development of cognitive, psychoaffective, and psychomotor aspects, as well as in its interface with language development^(2)^, as the child constructs an understanding of their own body and the world (cognitive dimension), experiences their feelings (psychoaffective dimension), and engages in social interactions and cultural integration^(3)^.

From Winnicott’s approach^(4)^, play is conceived as a creative process involving the interaction between the external world and the subjective world. In this way, play is perceived as the cultural manifestation of formations generated in the transitional space, which represents the process of separation as the child moves from absolute dependence to relative dependence on the mother, the latter being an expected position for the age range studied here^(4)^. Inspired by Winnicott^(4)^, several authors have observed signs of children’s psychic structuring at different ages^(5,6)^. They note that play provides fundamental clues for clinical work, including in Speech-Language Pathology^(1,3,7)^.

In its cognitive dimension, play also presents stages that, according to Piaget^(8)^, are necessary to reach the representational level, which, in the author’s view, would be fundamental for the emergence of language. In this sense, play also assumes an important role in its cognitive dimension within speech-language clinical practice^(3)^. This research focused on this dimension in its perspective on play. The analysis of play followed the classification proposed in the JASPER model by Casari et al.^(9)^. It comprises the following levels of play:

1. Simple - At this level, there are three sub-stages: indiscriminate use of objects with simple sensory exploration, discriminated use, and cause-and-effect play. This level predominates during the first year of life.
2. Combined - The child makes combinations according to the usual presentation of toys, as well as general combinations in which pieces from different toys are combined for play. This level typically emerges at around 12 months.
3. Pre-Symbolic - The child may engage in self-directed pretense and physical combinations; the child presents as an agent and may perform conventional combinations using some symbolic behavior in single-sequence scenes. This level typically emerges between 18 and 24 months.
4. Symbolic - The child represents concrete objects, pretends that one object is another, attributes life to inanimate objects, and reaches higher levels of abstraction and thought. This level is subdivided into sublevels: object substitution, substitution without objects, doll as agent, multi-scheme sequences, sociodramatization, and complex thematic play. It may emerge after 24 months and becomes more frequent and advanced between 36 and 48 months, a period during which narrative language development also expands.

The use of this classification was based on its clinical utility, as it allows an examination of spontaneous child play and aligns with the proposal through which the videos in this study’s database were produced.

Another important aspect to highlight is that, although the levels proposed by Casari et al.^(9)^ were developed within the JASPER model for children with Autism Spectrum Disorder (ASD), they are considered universal and allow for a unified and comparative perspective between that group of children and children with Developmental Language Disorder (DLD) and those with typical development.

DLD, previously known in the field of Speech-Language Pathology as “Specific Language Impairment” (SLI), was formalized through a 2014 publication in the International Journal of Language and Communication Disorders. The term DLD is defined by significant delays and persistent alterations in language acquisition, which may affect receptive language, expressive language, or both, in the absence of a developmental pathology that would account for such delay or alteration. In addition, language ability is below what is expected for chronological age, causing functional limitations in effective communication, social interaction, and school performance, individually or in any combination^(10,11)^. Currently, it is essential to carry out a differential diagnosis between DLD and ASD, considering the large number of children who arrive at clinical settings with the latter diagnosis. In this context, play may be fundamental, as previously mentioned, because it can provide essential signs regarding psychic structuring and cognitive development^(4-8)^.

The study by Mendes et al.^(12)^ analyzed symbolic maturity, intellectual performance, and vocabulary in children with DLD, showing that this group of children presented more primitive play, better receptive than expressive vocabulary, and average or above-average intelligence. These data underscore the importance of considering play in the therapeutic approach.

The study of play, together with other developmental assessment instruments such as the Dimensional Inventory of Child Development (IDADI)^(13)^ and the Labyrinth Scale^(14)^, may be complementary in establishing the differential diagnosis between ASD and DLD.

The IDADI^(13)^ is a parent-report protocol addressing developmental milestones across cognitive, socioemotional, receptive and expressive communication and language, gross and fine motor skills, and adaptive behavior. Although it includes some questions about play, it does not allow for the observation of spontaneous play. During the course of this research, it was observed that some parents report that the child engages in pretend play, whereas the child is still at the pre-symbolic level of play, which does not include this activity.

The Labyrinth Scale^(13)^ assesses core symptoms for the diagnosis of ASD. It encompasses social interaction skills, verbal and nonverbal communication, rigid behavior, and repetitive gestures. It consists of a parent or caregiver interview and a recorded interaction to assess the aforementioned aspects, among which spontaneous play is included. Although the Labyrinth Scale includes moments of proposed tasks for the child, especially regarding responses to joint attention and acceptance of proposed changes in play after a period of spontaneous play, it allows for the identification of baseline play through the initial observation of play that is entirely free. In addition, it offers a variety of toys that enable the child to demonstrate the levels proposed by Casari et al.^(9)^. The Labyrinth Scale includes an item on play; however, this item does not explore all the sublevels of analysis proposed in this research. Therefore, these instruments are understood to provide complementary perspectives.

It is known that children with ASD present more significant pragmatic and social difficulties than children with DLD, and that both groups present more communicative and social difficulties than children with typical development^(15)^, which is reflected in play. One study identified that children with some developmental disorder have a 9% lower likelihood of playing in general and play 17% less with their mothers than their typically developing peers^(16)^. These data highlight the importance of considering play as a space for intervention in developmental disorders in childhood. Although there are many studies reporting on language diagnosis and ASD cases^(12-18)^, there is a lack of studies specifically addressing play in cases of DLD, as few studies have examined this topic^(12,16)^.

The literature recognizes that language acquisition is a complex process, dependent on the interaction between neurobiological and social factors during child development. Several factors, such as prematurity and children’s linguistic and cognitive abilities, may negatively influence this process. In addition, variables such as maternal education and sociodemographic factors play important roles and may significantly impact language development in children^(19-21)^.

Motivated by these assumptions, this study’s objectives were to analyze play in children aged two to four years and eleven months with Developmental Language Disorder (DLD), and to investigate correlations between play, developmental dimensions, and the variables maternal education, family income, and gestational age.

## METHOD

This research is a cross-sectional quantitative study that is part of the research project “The relationship between delayed language acquisition and a history of psychological distress in children aged 2 to 4 years”, approved by the Research Ethics Committee (REC) of the Universidade Federal de Santa Maria under opinion no. 5,057,051, CAAE no. 52044121.6.0000.5346. Accordingly, all regulatory guidelines and standards for research involving human beings proposed by the Brazilian National Health Council in Resolutions 466/12 and 510/16 were followed. Participation in the study required that all guardians read and sign the Free and Informed Consent Term (FICT). They also received a confidentiality agreement signed by the principal investigator.

Data were collected by the researchers and composed an image database from which the analyses proposed in this study were carried out.

### Participants

This study’s sample consisted of images and assessment data from 37 children aged 2 years to 4 years and 11 months, diagnosed with Developmental Language Disorder (DLD), analyzed within the larger study previously mentioned.

### Assessment collection and analysis procedures

Children were selected from waiting lists or from extension projects or internships at the Speech-Language Pathology teaching clinics of the Federal Universities of Santa Maria and Rio Grande do Sul, considering their age range and complaints of delayed language acquisition.

After identifying delays in language acquisition, assessments were conducted to verify the diagnosis of DLD, including the Labyrinth Scale^(14)^, the Dimensional Inventory of Child Development (IDADI)^(13)^, among other tests deemed necessary by the speech-language pathologist researchers to establish the diagnosis of DLD^(22-24)^.

The Labyrinth Scale was used because it allows for differential diagnosis between DLD and Autism Spectrum Disorder (ASD). The IDADI, in turn, was used as a child development assessment tool to identify which dimensions of child development might be altered, particularly language. As an inclusion criterion for the study, children were required to present delays in the communication and receptive and/or expressive language dimensions of the IDADI. In the Labyrinth Scale, on the other hand, children could present alterations in verbal and nonverbal communication. However, as an exclusion criterion on the Labyrinth Scale, children could not reach the cutoff score for the other core symptoms of ASD.

The Labyrinth Scale was administered in its three stages: anamnesis, video recording of the in-person assessment, and video analysis. These stages were conducted by two certified researchers and reviewed by the Labyrinth team in cases of diagnostic uncertainty. Table 1 presents the cutoff scores of the Labyrinth Scale for the total score and its subscales.

**Table 1:** Total and Subscale Cutoff Scores of the Labyrinth Scale

Subscales	Cutoff score indicative of ASD
Social Interaction (SI)	≥3
Verbal Communication (VC)	≥4
Nonverbal Communication (NVC)	≥2
Rigid Behavior and Repetitive Gestures (RBRG)	≥4
Total Score	≥12

Source: Pondé et al.^(14)^

### Play assessment

Considering the classification of play proposed by Casari et al.^(9)^, videos from the Labyrinth Scale assessment were analyzed, and each child’s baseline play was identified, that is, the spontaneous play observed during most of the recording since, although several toys are made available, this assessment allows the child to choose and return to the play activity they most prefer. There is also an initial five-minute period during which the child explores the toys independently. These toys allowed for simple play, combined play, pre-symbolic play, and symbolic play. The assessment lasted an average of thirty minutes and was conducted by researchers with specific training in the Labyrinth Scale and in the JASPER play analysis.

Only videos in which children were able to engage in spontaneous play were included in the analysis of this study. Based on these recordings, the first author classified play in its cognitive dimension. This classification was reviewed by the researchers who collected the Labyrinth Scale data, including one author trained in the JASPER model.

### Classification of play used in the analysis

Based on the observation of the type of play and the child’s chronological age, a category was assigned to classify play as typical for the age range, delayed when it was one level below the expected play for the child’s age range, or significantly delayed when it was two or more levels below what is expected for the child’s age range. For this purpose, the play classification^(9)^ presented in Table 2 was considered.

**Table 2:** Analysis of play based on Casari et al.^(9)^

Expected age range	Level of Play
4 to 6 months	Sensorimotor exploration of objects and toys by putting them in the mouth, banging them, etc.
9 to 12 months	Explores the functioning of toys through simple intentional actions, such as rolling a ball, knocking down a tower, and action–reaction games.
12 months	Simple combination of toys.
18 to 24 months	Begins to play by reproducing everyday actions, displaying pre-symbolic behavior. Performs these actions on themselves and on dolls. Masters combined play with toys.
24 to 36 months	Masters pre-symbolic activities and begins to reach the symbolic level with simple narratives of thematic scenes.
36 to 59 months	Established symbolism with greater fantasy and dramatization.
60 months ahead	Board games with established rules played in groups.

To enable descriptive analysis, the analyzed data referred to children aged between 2 years and 4 years and 11 months. The classification up to the age range of 59 months was considered, according to the following criteria, in order to allow descriptive analysis:

Expected for the age group - when the child was at one of the levels expected for their age range. Between 24 and 36 months, the child should present at least pre-symbolic behaviors, and at 24 months some symbolic behaviors when closer to 36 months. Between 36 and 59 months, the child should have some level of established symbolism.

Delay by one level - when the child’s play was one level below what is expected for their age range; for example, if a child aged between 36 and 59 months presented pre-symbolic play, or if a child aged between 24 and 36 months presented combined play, and so forth.

Delayed by two or more levels - when a child aged between 24 and 36 months presented simple exploratory play, or when a child aged between 36 and 59 months presented combined play but had not yet reached the pre-symbolic level.

For statistical analysis, considering the small number of participants in the sample, the group was dichotomized into expected play development for the age range and delayed play development (one or more levels of delay in relation to the age range).

### Statistical analysis procedures

Descriptive and inferential analyses were performed using the JASP software. Descriptive analyses aimed to provide a concise summary of the data, which could be presented in numerical or graphical form. Inferential analyses were used to present a random sample of data collected from a population in another study in order to describe and make inferences about the population investigated in the present study.

For descriptive analyses, absolute and relative frequencies and percentages were used. For inferential analyses, the chi-square test (p<0.05) was calculated to compare model parameters, and Pearson’s correlation was applied to analyze the relationships among the variables studied.

## RESULTS

In Table 3, the sample is described in terms of age range and play categorization.

**Table 3:** Description of the sample, play, and communication and language

Person	Age	Classification of play considering age group	Play Description	Receptive Communication and Language IDADI	Expressive Communication and Language IDADI
1	3y5m	Appropriate for the age range	Symbolic – dramatization present using language.	Typical	Significant delay
2	3y	Delayed by one level	Pre-symbolic – physical combination.	Delay alert	Significant delay
3	2y11m	Delayed by one level	Pre-symbolic –conventional combinations.	Delay alert	Significant delay
4	2y11m	Delayed by one level	Pre-symbolic – self-directed pretend play.	Delay	Significant delay
5	4y9m	Delayed by two levels or more	Combinatory play – presentation combination.	Significant delay	Significant delay
6	3y11m	Appropriate for the age range	Symbolic – dramatization without language.	Typical	Significant delay
7	3y10m	Appropriate for the age range	Symbolic – dramatization present using language.	Delay	Significant delay
8	3y10m	Delayed by one level	Pre-symbolic – child as agent.	Delay	Significant delay
9	3y10m	Delayed by one level	Pre-symbolic – conventional combinations.	Delay	Significant delay
10	3y4m	Delayed by one level	Pre-symbolic – child as agent.	Delay	Significant delay
11	4y6m	Delayed by one level	Pre-symbolic – child as agent.	Delay	Typical
12	4y1m	Delayed by two levels or more	Pre-symbolic – child as agent.	Significant delay	Significant delay
13	3y	Delayed by two levels or more	Simple play – cause and effect.	Delay	Significant delay
14	3y9m	Delayed by one level	Pre-symbolic – child as agent.	Typical	Significant delay
15	3y9m	Delayed by one level	Pre-symbolic – child as agent.	Typical	Delay alert
16	2y	Delayed by one level	Pre-symbolic – child as agent.	Significant delay	Significant delay
17	3y8m	Delayed by one level	Simple play – cause and effect.	Delay alert	Delay alert
18	2y8m	Appropriate for the age range	Symbolic – multi-scheme sequence.	Typical	Significant delay
19	3y11m	Delayed by two levels or more	Simple play – cause and effect.	Significant delay	Significant delay
20	3y1m	Delayed by one level	Pre-symbolic play - pretending to be self-directed	Typical	Significant delay
21	3y8m	Delayed by one level	Pre-symbolic – child as agent.	Significant delay	Significant delay
22	3y1m	Delayed by one level	Pre-symbolic – child as agent.	Delay	Delay
23	2y4m	Delayed by one level	Combinatory play – presentation combination.	Typical	Significant delay
24	2y	Delayed by two levels or more	Simple play – cause and effect.	Typical	Delay alert
25	2y	Delayed by one level	Simple play – cause and effect.	Delay alert	Delay
26	4y4m	Delayed by one level	Pre-symbolic – child as agent sublevel.	Significant delay	Significant delay
27	4y2m	Delayed by one level	Pre-symbolic – child as agent.	Significant delay	Significant delay
28	2y4m	Delayed by one level	Combinatory play – presentation combination.	Typical	Significant delay
29	4y4m	Delayed by one level	Pre-symbolic – child as agent.	Significant delay	Delay
30	2y9m	Delayed by one level	Pre-symbolic play - pretending to be self-directed	Typical	Significant delay
31	2y7m	Delayed by two levels or more	Combinatory play – presentation combination.	Typical	Significant delay
32	3y4m	Delayed by one level	Pre-symbolic – child as agent.	Typical	Delay alert
33	2y5m	Delayed by one level	Pre-symbolic – child as agent.	Typical	Significant delay
34	3y2m	Delayed by one level	Pre-symbolic play - pretending to be self-directed	Delay alert	Significant delay
35	2y11m	Delayed by one level	Pre-symbolic – child as agent.	Delay	Delay
36	2y4m	Appropriate for the age range	Pre-symbolic – child as agent.	Typical	Significant delay
37	3y7m	Delayed by one level	Pre-symbolic – child as agent.	Typical	Delay alert

**Caption:** a = year; m = month; IDADI = Dimensional Inventory of Child Development

**Figure 1:**
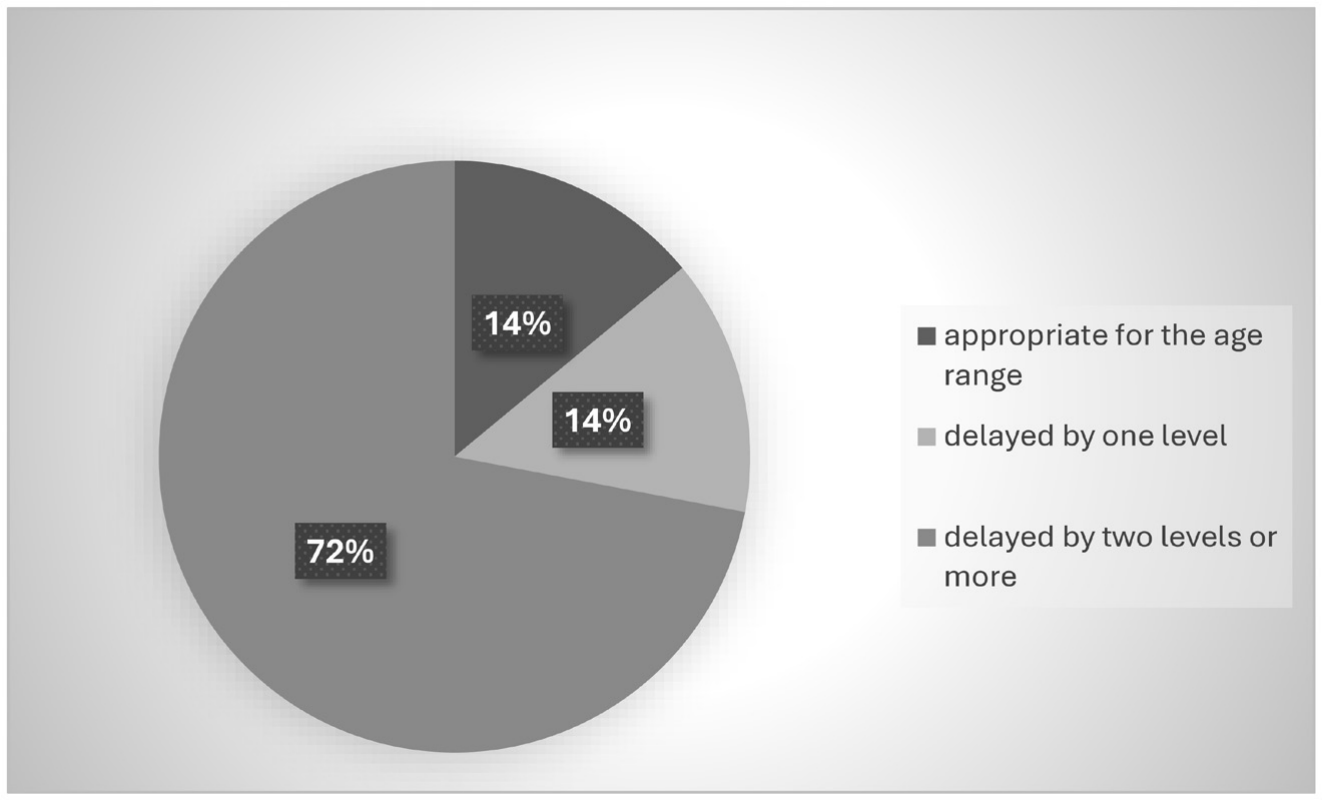
Descriptive percentage of play in the sample

Most children presented a delay of one level or more in play, as shown in Figure 1.

**Figura 2:**
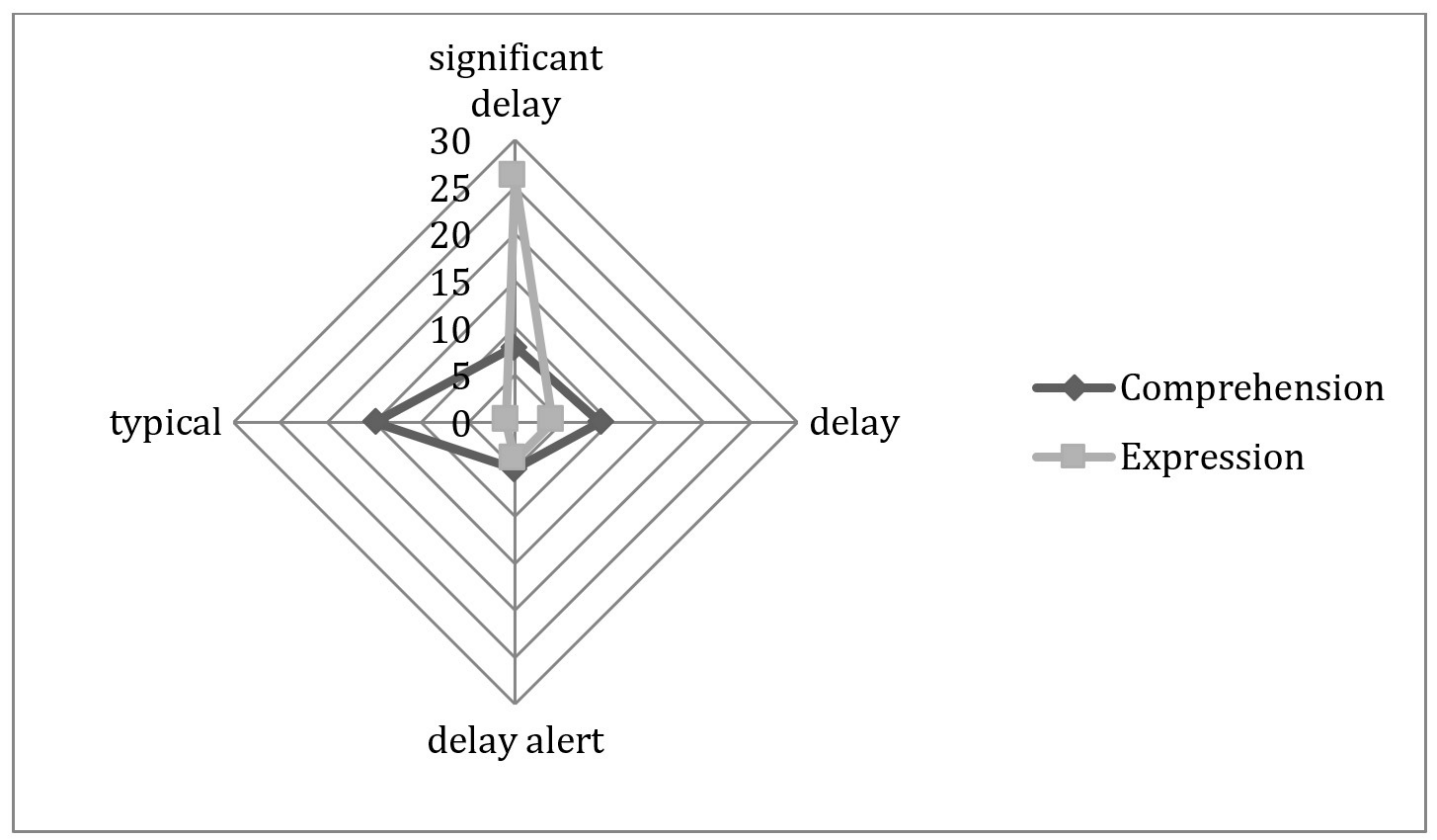
IDADI - Communication and Language

Regarding receptive and expressive communication and language, most children presented a significant delay in expressive communication and language and typical development in comprehension, as shown in Figure 2.

In the sample of 37 participants, of whom 24 were boys and 13 were girls, it was observed that most of the sample consisted of late preterm children, with very similar family income and maternal education across the group, as shown in Table 4. Statistical analysis found no association between play, gestational age, and family income.

**Table 4:** Description of gestational age, family income, and maternal education

	**Categorization**	**Number (%)**
**Gestational Age**	Less than 32 weeks	1 (3)
	32 to 36 weeks	27 (79)
	37 to 41 weeks	6 (18)
**Maternal Education**	Elementary Education	1 (2.6)
	High School	12 (31.5)
	Higher Education	25 (65.7)
**Family Income**	Up to 1 minimum wage	2 (7.6)
	Between 1 and 2 minimum wages	2 (7.6)
	Between 2 and 3 minimum wages	4 (15.3)
	Between 3 and 5 minimum wages	4 (15.3)
	Between 5 and 10 minimum wages	9 (34.6)
	More than 10 minimum wages	5 (19.2)

Table 5 presents the results of the analysis of the association between the IDADI developmental dimensions and play.

**Table 5:** Analysis of the association between play and IDADI

	Play classification		p-value
IDADI-Fine motor skills	Appropriate AR	Delay	0.035
	N(%)	N(%)	
Typical	3 (60)	26 (78)	
Delay alert	1 (20)	2 (6)	
Delay	1 (20)	0 (0)	
Significant delay	0 (0)	5 (15)	
IDADI-Cognition			0.63
Typical	2 (40)	12 (36)	
Delay alert	2 (40)	8 (24)	
Delay	1 (20)	5 (15)	
Significant delay	0 (0)	8 (24)	
IDADI-Socioemotional			0.19
Typical	2 (40)	18 (54)	
Delay alert	3 (60)	6 (18)	
Delay	0 (0)	6 (18)	
Significant delay	0 (0)	3 (9)	
IDADI-Receptive communication and language			0.19
Typical	4 (80)	11 (33)	
Delay alert	0 (0)	7 (21)	
Delay	1 (20)	7 (21)	
Significant delay	0 (0)	8 (24)	
IDADI-Communication and expressive language			0.56
Typical	0(0)	1(3)	
Delay alert	0(0)	5(15)	
Delay	0(0)	4(12)	
Significant delay	5 (100)	23 (69)	
IDADI – Gross motor skills			0.61
Typical	5 (100)	24 (72)	
Delay alert	0 (0)	4 (12)	
Delay	0 (0)	3 (9)	
Significant delay	0 (0)	2 (6)	
IDADI – Adaptive behavior			0.91
Typical	3 (60)	19 (57)	
Delay alert	1 (20)	6 (18)	
Delay	1 (20)	5 (15)	
Significant delay	0 (0)	3 (9)	

**Caption:** IDADI = Dimensional inventory of child development; AR = Age Range

## DISCUSSION

The analysis of the results highlights the importance of play assessment among children with DLD. The relevance of assessing communication and language dimensions for therapeutic planning is well established; however, play, as a space for interaction with children during therapeutic sessions, plays a fundamental role in the practice of the speech-language pathologist^(21,25)^.

The study by Graña and Ramos^(7)^ observed that the speech-language pathologist’s playful activity has effects on therapeutic practice. The results of the present study highlight the importance of speech-language pathologists analyzing the play of the children they treat in order to develop comprehensive therapeutic planning that includes play as a foundation for symbolism, as several studies have already suggested in childhood clinical practice^(1-7,21,25,26)^. It also emphasizes that spontaneous play is far more effective in anchoring language development than attempts to disguise speech exercises through directed play^(7)^.

The analysis of Table 3 highlights a delay of one level or more in play in 35 of the 37 children studied. Most of these children (36) presented a significant delay in expressive communication and language as analyzed through the IDADI, and only one child presented a delay in comprehension. Thus, among the children diagnosed with DLD by the speech-language pathologists, there was a significant delay in play together with language delay, although this was not statistically evidenced. It would be of interest for future studies to compare a larger number of children with typical language development, without DLD, and children with DLD in order to verify whether a statistical association between language and play might emerge.

Similarly, it is hypothesized that no statistical association was found between play and other developmental dimensions, except for fine motor development, because there were no alterations and variations that produced statistical differences in the studied group that could be evidenced in relation to play. This result was similar to that found in the study by Mendes et al.^(12)^, which also did not identify an association between symbolic maturity, assessed in a manner similar to how play was evaluated in the present study, and variables such as vocabulary and intellectual performance.

In fine motor skills, however, greater variation was observed, which may be associated with the fact that some children in the sample presented expressive language difficulties related to phonetic-phonological or articulatory skills. The interface between DLD and late prematurity found in the sample may explain the motor delays, also reported in other studies^(18,19)^.

Regarding gestational age, despite the absence of statistical correlation, it should also be noted that most of the sample consisted of late preterm children, thus showing little variability within the sample. One aspect that should be emphasized, however, concerns the possible effects of late prematurity on language development, as the literature has extensively discussed the effects of extreme prematurity and other factors such as low birth weight and length of hospital stay, among others^(21)^. Nevertheless, the descriptive data from this study suggest that late preterm children deserve careful attention regarding their development and language acquisition.

The literature describes language acquisition as a complex process that depends on the proper functioning of encephalic and cognitive structures, constituting an intricate developmental process, in which any impairment in one of these elements may exert a significant impact on child development^(18,19,27)^. In this context, it is worth noting that in Brazil, approximately 340,000 babies are born preterm each year, which corresponds to 931 per day or six preterm births every 10 minutes, with more than 12% of births in the country occurring before 37 weeks of gestation, representing a rate twice as high as that observed in European countries^(28)^. Thus, prematurity emerges as a biological risk factor associated with possible alterations in child language development and should be considered in the monitoring of children.

Concerning maternal education and family income, once again, given that most mothers had completed at least high school education and had family incomes above five minimum wages, the effects of these sociodemographic aspects may not have been evident in the sample, despite being strongly emphasized in other studies, such as one^(20)^ that correlates language acquisition with maternal education and shows that families with low income may present approximately 50% delay in development, even after adjustments for maternal education.

## CONCLUSION

Considering this research's initial objectives, the descriptive analysis showed that children with Developmental Language Disorder presented delayed play for their age range, and that better play development was positively associated with better fine motor performance in the studied sample.

The absence of statistical associations with other developmental dimensions and variables such as family income, gestational age, and maternal education suggests a degree of homogeneity and more favorable conditions for these aspects within the studied group. Nevertheless, it is important to consider that a large number of children in the sample were late preterm, which may have acted as a trigger for the manifestation of DLD, delayed play, and its association with fine motor alterations. This finding suggests that late preterm children, similar to those born extremely preterm, also require differentiated follow-up in routine child health care.

Some study’s limitations should be highlighted, as data collection occurred during the pandemic period, which did not allow for a more extensive assessment of play or a qualitative evaluation of language. Even so, based on the researchers’ experience, it was possible to obtain a reliable sample of video recordings for the analysis of play, considering the procedures established for the administration of the Labyrinth Scale. The study confirms that play is a fundamental topic in the clinical practice of language disorders and should continue to be investigated in larger samples in future quantitative studies. It also suggests that qualitative studies and investigations of the psychic dimension of play should be conducted, as alterations in symbolism need to be explored in their full scope.
