# Supplementary material for: Analysis of play in children aged 2 to 4 with developmental language disorder
Source: Codas. 2026 Apr 10;38(2):e20240098. doi: 10.1590/2317-1782/e20240098en (PMC13075841; doi:10.1590/2317-1782/e20240098en)
Supplement: Translation

# Translation: Análise do brincar de crianças de 2 a 4 anos com transtorno do desenvolvimento da linguagem

**DOI:** 10.1590/2317-1782/e20240098pt

**Authors:** Júlia França Viana, Tainá Rossato Benfica, Gustavo Marcelino Siquara, Ana Paula Ramos de Souza

**Affiliations:** 1 Departamento de Saúde e Comunicação Humana, Universidade Federal do Rio Grande do Sul – UFRGS - Porto Alegre (RS), Brasil.; 2 Programa de Pós-graduação em Distúrbios da Comunicação Humana, Universidade Federal de Santa Maria – UFSM - Santa Maria (RS), Brasil.; 3 Programa de Pós-graduação em Medicina e Saúde Humana, Escola Bahiana de Medicina – EBM - Salvador (BA), Brasil.

**Keywords:** Jogos e Brinquedos, Desenvolvimento Infantil, Fatores Socioeconômicos, Transtorno do Desenvolvimento da Linguagem, Idade Gestacional

## Abstract

**Objetivo:**

Analisar o brincar em crianças de dois a quatro anos e onze meses de idade, com Transtorno do Desenvolvimento da Linguagem (TDL). Investigar correlações entre o brincar, dimensões do desenvolvimento e as variáveis escolaridade materna, renda familiar e idade gestacional.

**Método:**

Trata-se de uma pesquisa quantitativa e transversal, com 37 crianças na faixa etária de dois a quatro anos e onze meses, diagnosticadas por fonoaudiólogas com TDL. Este diagnóstico foi confirmado a partir de dois instrumentos: Escala Labirinto que permite uma avaliação diferencial entre TDL e Transtorno do Espectro do Autismo (TEA) e o Inventário Dimensional de Desenvolvimento Infantil (IDADI) que permite confirmar o atraso no desenvolvimento da linguagem, bem como avaliar outras dimensões do desenvolvimento infantil. Avaliou-se o brincar por meio da análise da filmagem da interação entre criança e pesquisador durante coleta da Escala Labirinto, considerando níveis esperados por idade cronológica de dois a cinco anos. A partir das categorias identificadas foram feitas análises descritivas e estatísticas, das variáveis escolaridade materna, idade gestacional e renda familiar.

**Resultados:**

A maior parte das crianças apresentou atraso de um nível ou mais no desenvolvimento do brincar, acompanhado de atraso significativo na linguagem expressiva. Houve associação estatística entre o brincar e a dimensão motora fina do desenvolvimento infantil.

**Conclusão:**

Crianças com TDL evidenciaram atraso no brincar e o melhor desenvolvimento neste se associa positivamente ao melhor desempenho em motricidade fina, o que sugere sua relevância enquanto espaço de desenvolvimento cognitivo e de subjetivação.

## INTRODUÇÃO

É indubitável que o ato de brincar é amplamente reconhecido como fundamental para o desenvolvimento infantil. A prática do brincar é considerada como uma prática universal que exibe características distintas dentro dos contextos social, histórico e cultural^(1,2)^. Nesse sentido, o brincar assume um papel significativo no desenvolvimento de aspectos cognitivo, psicoafetivo, psicomotor e na interface com o desenvolvimento da linguagem^(2)^, pois a criança constrói o entendimento sobre o próprio corpo e o mundo (dimensão cognitiva), além de vivenciar seus sentimentos (dimensão psicoafetiva), e vivenciar interações sociais e integração cultural^(3)^.

Na abordagem de Winnicott^(4)^, o brincar é concebido como um processo criativo que envolve a interação entre o mundo externo e o subjetivo. Dessa maneira, a brincadeira é percebida como a manifestação cultural de formações geradas no espaço transicional, que representa o processo de separação, na saída da dependência absoluta para a dependência relativa da criança em relação à mãe, sendo esta última uma posição esperada para a faixa etária aqui estudada^(4)^. Inspirados em Winnicott^(4)^, diversos autores observaram sinais da estruturação psíquica da criança em distintas idades^(5,6)^. Observam que o brincar fornece pistas fundamentais ao trabalho clínico, também na Fonoaudiologia^(1,3,7)^.

Em sua dimensão cognitiva, o brincar também apresenta etapas que, segundo Piaget^(8)^, são necessárias para chegar ao nível representativo, o que seria fundamental, na visão do autor, para se chegar à linguagem. Nesse sentido, o brincar assume um papel importante também em sua dimensão cognitiva na clínica fonoaudiológica^(3)^.Esta pesquisa enfocou esta dimensão em seu olhar sobre o brincar. A análise do Brincar seguiu a classificação proposta no modelo JASPER por Casari et al.^(9)^. Ela abrange os seguintes níveis de jogo:

1. Simples – Neste nível existem três subetapas: o uso indiscriminado do objeto com simples exploração sensorial, o uso discriminado e os jogos de causa-efeito. Este nível domina o primeiro ano de vida.
2. Combinado- a criança faz combinações de acordo com a apresentação usual dos brinquedos, e a combinação geral em que combina peças de diferentes brinquedos para jogar. Esse nível costuma emergir aos 12 meses.
3. Pré-Simbólico- a criança pode fingir autodirigido, combinação física, a criança se apresenta como agente, pode fazer combinações convencionais usando alguma conduta simbólica em cenas de sequência única. Costuma emergir entre 18 e 24 meses.
4. Simbólico- A criança faz a representação dos objetos concretos, finge que um objeto é outra coisa, atribui vida ao inanimado, atingindo níveis maiores de abstração e pensamento. O nível é subdividido em subníveis: substituição com objeto, substituição sem objeto, boneco como agente, sequência multiesquema, sociodramatização e brinquedo temático complexo. Pode emergir após 24 meses sendo mais frequente e evoluído entre 36 e 48 meses, período em que o desenvolvimento narrativo da linguagem também se amplia.

A utilização desta classificação se deu por sua utilidade clínica na medida que permite um olhar sobre o brincar espontâneo infantil e se combina com a proposta por meio da qual foram feitos os vídeos do banco de dados desta pesquisa.

Outro aspecto importante a ressaltar que, embora os níveis de Casari et al.^(9)^ tenham sido propostos no modelo JASPER para crianças com Transtorno do Espectro Autista (TEA), considera-se que sejam universais e permitam um olhar unificado e comparativo entre aquele grupo de crianças e as crianças com Transtorno do Desenvolvimento de Linguagem (TDL) e com desenvolvimento típico.

O TDL, conhecido anteriormente no campo de Fonoaudiologia como “Distúrbio Específico da Linguagem” (DEL), foi oficializado por meio da publicação em 2014 no *International Journal of Language and Communication Disorders*. O termo TDL ficou definido por significativos atrasos e alterações persistentes na aquisição da linguagem que podem acometer tanto o domínio linguístico receptivo, expressivo ou ambos, na ausência de patologia do desenvolvimento que desencadeia tal atraso ou alteração. Além disso, na capacidade linguística abaixo do esperado para idade cronológica, causando limitações funcionais na comunicação efetiva, na interação social, no desempenho na escola, individualmente ou em qualquer combinação^(10,11)^. Na atualidade, torna-se fundamental realizar o diagnóstico diferencial entre TDL e TEA, considerando o grande número de crianças que chegam à clínica com este último diagnóstico. Nesse sentido, o brincar pode ser fundamental, pois, conforme já mencionado, ele pode fornecer sinais fundamentais sobre a estruturação psíquica e a evolução cognitiva^(4-8)^.

O estudo de Mendes et al.^(12)^ analisou a maturidade simbólica, desempenho intelectual e o vocabulário de crianças com TDL, evidenciando que este grupo de crianças apresentaram jogos mais primitivos, com melhor vocabulário receptivo do que expressivo e inteligência na média ou superior. Esses dados ressaltam a importância de considerar o brincar na abordagem terapêutica.

O estudo do brincar, em conjunto com outros instrumentos de avaliação do desenvolvimento como o Inventário Dimensional de Avaliação do Desenvolvimento Infantil (IDADI)^(13)^ e a Escala Labirinto^(14)^, podem ser complementares na efetivação do diagnóstico diferencial entre TEA e TDL.

O IDADI^(13)^ é um protocolo sobre marcos evolutivos nas dimensões cognitiva, socioemocional, comunicação e linguagem receptiva e expressiva, motricidade ampla e fina, e comportamento adaptativo, preenchido pelos pais, que possui algumas questões sobre o brincar mas não permite a visualização do brincar espontâneo. Observou-se, durante a realização desta pesquisa, que há pais que dizem que a criança faz de conta mas a criança ainda estaria no brincar pré-simbólico que não inclui esta atividade.

A Escala Labirinto^(13)^ avalia os sintomas centrais para o diagnóstico de TEA. Esta abrange habilidades de interação social, comunicação verbal e não verbal, comportamento rígido e gestos repetitivos. Ela consta de uma anamnese com os pais ou responsáveis e uma filmagem de interação para avaliação dos aspectos citados, entre os quais o brincar espontâneo é um deles. Embora a Escala Labirinto tenha momentos de proposição de consignas para a criança, sobretudo em relação à resposta à atenção compartilhada e à aceitação da proposição de mudança de brincadeira após um tempo de brincar espontâneo da criança, ela permite que se encontre o brincar de base a partir da observação inicial do brincar que é totalmente livre. Além disso, ela propõe brinquedos variados que permitem que a criança evidencie os níveis propostos por Casari et al.^(9)^. A Escala Labirinto possui um item sobre o brincar, no entanto, esse item não explora todos os subníveis de análise propostos nesta pesquisa. Por isso, entende-se que possam ser visões complementares.

Sabe-se que crianças com TEA apresentaram dificuldades pragmáticas e sociais mais significativas do que as crianças com TDL, e que ambos grupos apresentam mais dificuldades comunicativas e sociais do que as crianças com desenvolvimento típico^(15)^, o que se reflete no brincar. Um estudo identificou que crianças com algum distúrbio de desenvolvimento possuem 9% menos chance de brincar em geral e que brincam 17% menos com suas mães do que seus pares com desenvolvimento típico^(16)^. Esses dados identificam a importância de pensar no brincar enquanto um espaço de intervenção nos distúrbios do desenvolvimento infantil. Embora haja muitos estudos relatados sobre o diagnóstico de linguagem e nos casos de TEA^(12-18)^, há carência de estudos específicos sobre o brincar nos casos de TDL, já que poucos estudos abordam o tema^(12,16)^.

A literatura reconhece que a aquisição da linguagem é um processo complexo, dependente da interação entre fatores neurobiológicos e sociais durante o desenvolvimento infantil. Diversos fatores, como prematuridade, habilidades linguísticas e cognitivas das crianças, podem influenciar negativamente esse processo. Além disso, variáveis como a escolaridade materna e fatores sociodemográficos desempenham papéis importantes e podem impactar significativamente o desenvolvimento da linguagem na criança^(19-21)^.

Motivada por tais pressupostos, os objetivos desta pesquisa foram analisar o brincar em crianças de dois a quatro anos e onze meses de idade, com Transtorno do Desenvolvimento da Linguagem (TDL). Investigar correlações entre o brincar, dimensões do desenvolvimento e as variáveis escolaridade materna, renda familiar e idade gestacional.

## MÉTODO

A presente pesquisa trata-se de um estudo do tipo quantitativo transversal que se insere no projeto de pesquisa "A relação entre atraso na aquisição da linguagem e histórico de sofrimento psíquico em crianças na faixa etária de 2 e 4 anos” aprovado pelo Comitê de Ética em Pesquisa da Universidade Federal de Santa Maria sob parecer 5.057.051, com número de CAAE 52044121.6.0000.5346. Dessa forma, foram cumpridas todas as diretrizes e normas regulamentadoras para pesquisa envolvendo seres humanos propostas pelo Conselho Nacional de Saúde em suas resoluções 466/12 e 510/16. A participação na pesquisa exigia que todos os responsáveis realizassem a leitura e assinatura do Termo de Consentimento Livre e Esclarecido (TCLE). Os mesmos receberam o termo de confidencialidade assinado pela pesquisadora responsável.

Os dados foram coletados pelas pesquisadoras e compuseram um banco de dados de imagens a partir do qual foi realizada a análise proposta nesta pesquisa.

### Participantes

A amostra deste estudo foi composta pelas imagens e dados de avaliações de 37 crianças, na faixa etária de 2 a 4 anos e 11 meses, com diagnóstico de TDL analisadas na pesquisa maior já referida.

### Procedimentos de coleta e análise das avaliações

As crianças foram selecionadas nas listas de espera ou nos projetos de extensão ou estágios das clínicas-escola de Fonoaudiologia das Universidades Federais de Santa Maria e do Rio Grande do Sul, considerando a sua faixa etária e a queixa de atraso na aquisição da linguagem.

A partir da identificação do atraso na aquisição da linguagem, foram realizadas avaliações para verificar o diagnóstico de TDL, entre as quais: a Escala Labirinto^(14)^, o Inventário Dimensional de Desenvolvimento Infantil (IDADI)^(13)^, entre outros testes que os fonoaudiólogos pesquisadores julgaram necessários para a realização do diagnóstico de TDL^(22-24)^.

A Escala Labirinto foi utilizada porque permite o diagnóstico diferencial entre TDL e TEA. Já o IDADI foi utilizado como teste de avaliação do desenvolvimento infantil de modo a identificar quais dimensões do desenvolvimento infantil poderiam estar alteradas, sobretudo a linguagem. Como critério de inclusão na pesquisa, era necessário que as crianças apresentassem atraso nas dimensões de comunicação e linguagem compreensiva e/ou expressiva do IDADI. Na Escala Labirinto, por outro lado, as crianças poderiam apresentar alterações na comunicação verbal e não verbal. Contudo, como critério de exclusão na Escala Labirinto, as crianças não poderiam atingir o ponto de corte nos demais sintomas centrais para TEA.

A Escala Labirinto foi coletada em suas três etapas: realização da anamnese, filmagem da coleta presencial e análise do vídeo. Essas etapas foram realizadas pelas duas pesquisadoras certificadas e conferidas pela equipe da Labirinto em caso de dúvida diagnóstica. Na Tabela 1, são expostos os pontos de corte da Escala Labirinto para a pontuação total e suas subescalas.

**Tabela 1:** Ponto de corte total e das subescalas da Escala Labirinto

Subescalas	Ponto de corte para indicativo de TEA
Interação Social (IS)	≥3
Comunicação Verbal (CV)	≥4
Comunicação Não Verbal (CNV)	≥2
Comportamento Rígido e Gesto Repetitivo (CRGR)	≥4
Pontuação Total	≥12

Fonte: Pondé et al.^(14)^

### Avaliação do brincar

Considerando a classificação do brincar proposta por Casari et al.^(9)^, os vídeos da avaliação com a Escala Labirinto foram analisados e foi considerado o brincar de base de cada criança, ou seja, aquele brincar espontâneo na maior parte do tempo da filmagem, já que, embora sejam disponibilizados vários brinquedos, esta avaliação permite que a criança escolha e volte à brincadeira que mais deseje. Também há um tempo inicial de cinco minutos em que a criança explora os brinquedos sozinha. Esses brinquedos continham a possibilidade de jogos simples, compostos, brincar pré-simbólico e simbólico. A aplicação durou, em média, trinta minutos e foi coletada pelas pesquisadoras com formação específica para a Escala Labirinto e na análise do brincar do JASPER.

Só foram computadas para análise nesta pesquisa, os vídeos em que as crianças conseguiram brincar espontaneamente. A partir dessas filmagens, a primeira autora realizou a classificação do brincar, em sua dimensão cognitiva. Essa classificação foi conferida pelas pesquisadoras que coletaram os dados da Escala Labirinto, entre as quais uma autora com formação no modelo JASPER.

### Classificação do brincar utilizada na análise

A partir da visualização do tipo de brincadeira e da idade da cronológica da criança foi atribuída uma categoria quanto ao brincar ser típico para faixa etária ou estar atrasado quando estava um nível abaixo do brincar da faixa etária da criança ou atrasado significativamente, quando estava dois ou mais níveis abaixo do esperado para a faixa etária da criança. Para tanto, considerou-se a classificação do brincar^(9)^ prevista na Tabela 2.

**Tabela 2:** Análise do Brincar baseada em Casari et al.^(9)^

Faixa etária esperada	Nível do brincar
4 a 6 meses	Exploração sensório-motora de objetos e brinquedos colocando-os na boca, batendo, etc.
9 a 12 meses	Explora o funcionamento dos brinquedos com ações simples intencionais como rolar uma bola, derrubar uma torre e jogos de ação e reação.
12 meses	Combinação simples de brinquedos.
18 a 24 meses	Começa a brincar reproduzindo ações do cotidiano, em conduta pré-simbólica.Realiza essas ações em si mesma e nos bonecos. Domina o jogo composto com brinquedos.
24 a 36 meses	Domina atividades pré-simbólicas e começa a atingir o nível simbólico com narrativas simples de cenas temáticas.
36 a 59 meses	Simbolismo estabelecido com maior fantasia e dramatização.
60 meses em diante	Jogos de mesa com regras estabelecidas em grupo.

Para viabilizar a análise descritiva, os dados analisados eram de crianças na faixa etária entre 2 anos e 4 anos e 11 meses, considerou-se a classificação até a faixa etária de 59 meses, a partir da seguinte classificação para possibilitar a análise descritiva:

Esperado para a faixa etária- quando a criança estivesse em algum dos níveis esperados para sua faixa etária. Entre 24 e 36 meses ter condutas ao menos pré- simbólicas aos 24 meses algumas condutas simbólicas quando mais próxima aos 36 meses. Entre 36 e 59 meses a criança deveria estar com algum nível de simbolismo estabelecido.

Atraso um nível- quando a criança estivesse com o brincar um nível abaixo do esperado para sua faixa etária, por exemplo, se na faixa de entre 36 e 59 meses estivesse com o brincar pré-simbólico, se na faixa entre 24 e 36 meses com o jogo combinado e assim por diante.

Atraso dois níveis ou mais- se uma criança entre 24 e 36 meses estivesse no exploratório simples ou ainda se uma criança entre 36 e 59 meses estivesse no jogo combinatório composto, mas ainda não estivesse no pré-simbólico.

Para a análise estatística, considerando o número pequeno de sujeitos da amostra, o grupo foi diferenciado entre desenvolvimento do brincar esperado para a faixa etária e atrasado (um ou mais níveis de atraso em relação à faixa etária).

### Procedimentos de análise estatística

Os procedimentos de análises descritivas e inferenciais foram realizados por meio do software JASP. As análises descritivas têm como objetivo fornecer um resumo conciso dos dados que podem ser apresentados de forma numérica ou gráfica. As análises inferenciais foram utilizadas para apresentar uma amostra aleatória dos dados coletados de uma população em uma outra pesquisa para descrever e fazer inferências sobre a atual população pesquisada.

Para as análises descritivas foi utilizada frequência absoluta e relativa e percentagem. Para as análises inferenciais, calculou-se o qui-quadrado (p<0,05) a fim de comparar os parâmetros do modelo e correlação de Pearson com o objetivo de analisar as relações entre as variáveis estudadas.

## RESULTADOS

Na Tabela 3, há uma descrição da amostra em termos da faixa etária e categorização do brincar.

**Tabela 3:** Descrição da amostra, do brincar, da comunicação e linguagem

Sujeito	Idade	Classificação do brincar considerando Faixa Etária	Descrição do Brincar	Comunicação e Linguagem Compreensiva IDADI	Comunicação e Linguagem Expressiva IDADI
1	3a5m	Adequado à FE	Simbólico dramatização presente usando a linguagem.	Típica	Atraso significativo
2	3a	Atraso um nível	pré-simbólico - combinação física.	Alerta de atraso	Atraso significativo
3	2a11m	Atraso um nível	pré-simbólico - combinações convencionais.	Alerta de atraso	Atraso significativo
4	2a11m	Atraso um nível	pré-simbólico- fingir autodirigido.	Atraso	Atraso significativo
5	4a9m	Atraso dois níveis ou mais	jogo combinatório - combinação de apresentação.	Atraso significativo	Atraso significativo
6	3a11m	Adequado à FE	simbólico dramatização sem linguagem	Típica	Atraso significativo
7	3a10m	Adequado à FE	simbólico dramatização presente usando a linguagem	Atraso	Atraso significativo
8	3a10m	Atraso um nível	pré-simbólico -criança como agente	Atraso	Atraso significativo
9	3a10m	Atraso um nível	pre-simbólico - combinações convencionais.	Atraso	Atraso significativo
10	3a4m	Atraso um nível	pré-simbólico - criança como agente.	Atraso	Atraso significativo
11	4a6m	Atraso um nível	Pré-simbólico- criança como agente.	Atraso	Típica
12	4a1m	Atraso dois níveis ou mais	pré-simbólico - criança como agente.	Atraso Significativo	Atraso significativo
13	3a	Atraso dois níveis ou mais	jogo simples - causa efeito.	Atraso	Atraso significativo
14	3a9m	Atraso um nível	Pré-simbólico – criança como agente.	Típica	Atraso significativo
15	3a9m	Atraso de um nível	Pré-simbólico – criança como agente.	Típica	Alerta de atraso
16	2a	Atraso de um nível	Pré-simbólico – criança como agente.	Atraso significativo	Atraso significativo
17	3a8m	Atraso em um nível	Jogo simples - causa e efeito	Alerta atraso	Alerta de atraso
18	2a8m	Adequado à FE	Simbólico – sequência multiesquema.	Típica	Atraso significativo
19	3a11m	Atraso dois níveis ou mais	Jogo simples - causa e efeito	Atraso significativo	Atraso significativo
20	3a1m	Atraso um nível	Pré-simbólico - fingir autodirigido	Típica	Atraso significativo
21	3a8m	Atraso um nível	Pré-simbólico - criança como agente	Atraso significativo	Atraso significativo
22	3a1m	Atraso de um nível	Pré-simbólico - criança como agente	Atraso	Atraso
23	2a4m	Atraso um nível	Jogo combinatório- combinação de apresentação	Típica	Atraso significativo
24	2a	Atraso dois níveis ou mais	jogo simples - causa efeito	Típica	Alerta de atraso
25	2a	Atraso um nível	Jogo simples - causa e efeito.	Alerta de atraso	Atraso
26	4a4m	Atraso de um nível	pré-simbólico. Subnível criança como agente	Atraso significativo	Atraso significativo
27	4a2m	Atraso um nível	Pré-simbólico - criança como agente	Atraso significativo	Atraso significativo
28	2a4m	Atraso um nível	Jogo Combinatório - Combinação de apresentação.	Típica	Atraso significativo
29	4a4m	Atraso um nível	pré-simbólico - criança como agente	Atraso significativo	Atraso
30	2a9m	Atraso um nível	Pré-simbólico - fingir autodirigido.	Típica	Atraso significativo
31	2a7m	Atraso dois níveis ou mais	Jogo Combinatório - Combinação de apresentação	Típica	Atraso significativo
32	3a4m	Atraso um nível	Pré-simbólico - criança como agente	Típica	Alerta de atraso
33	2a5m	Atraso um nível	Pré-simbólico - criança como agente	Típica	Atraso significativo
34	3a2m	Atraso um nível	Pré-simbólico - fingir autodirigido	Alerta de atraso	Atraso significativo
35	2a11m	Atraso um nível	Pré-simbólico - criança como agente	Atraso	Atraso
36	2a4m	Adequado à FE	Pré-simbólico - criança como agente	Típica	Atraso significativo
37	3a7m	Atraso um nível	Pré-simbólico - criança como agente	Típica	Alerta de atraso

**Legenda:** a = ano; m =mês; IDADI = Inventário dimensional do desenvolvimento infantil

A maior parte das crianças possui atraso de um nível ou mais no brincar, como se vê na Figura 1.

**Figura 1:** Percentual descritivo do brincar na amostra

Em relação à comunicação e linguagem receptiva e expressiva a maior parte possui atraso significativo na comunicação e linguagem expressiva e desenvolvimento típico na compreensão, como se pode ver na Figura 2.

**Figura 2:** IDADI-Comunicação e Linguagem

Na amostra de 37 sujeitos, dos quais 24 eram meninos e 13 meninas, observou-se que a maior parte da amostra era constituída por prematuros tardios, com renda familiar e escolaridade materna muito similar no grupo, como se vê na Tabela 4. Na análise estatística não foi encontrada associação entre o brincar, a idade gestacional e a renda familiar.

**Tabela 4:** Descrição da idade gestacional, renda familiar e escolaridade materna

	**Categorização**	**Número (%)**
**Idade Gestacional**	Menos 32 semanas	1 (3)
	32 a 36 semanas	27 (79)
	37 a 41 semanas	6 (18)
**Escolaridade Materna**	Fundamental	1 (2,6)
	Médio	12 (31,5)
	Superior	25 (65,7)
**Renda Familiar**	Até 1 salário-mínimo	2 (7,6)
	Entre 1 e 2 salários-mínimos	2 (7,6)
	Entre 2 e 3 salários-mínimos	4 (15,3)
	Entre 3 e 5 salários-mínimos	4 (15,3)
	Entre 5 e 10 salários-mínimos	9 (34,6)
	Acima de 10 salários-mínimos	5 (19,2)

Na Tabela 5 estão colocados os resultados da análise da associação entre as dimensões do desenvolvimento do IDADI e o brincar.

**Tabela 5:** Análise da associação entre o brincar e o IDADI

	Classificação do brincar		p-valor
IDADI-Motricidade fina	Adequado FE	Atraso	0,035
	N(%)	N(%)	
Típico	3 (60)	26 (78)	
Alerta de atraso	1 (20)	2 (6)	
Atraso	1 (20)	0 (0)	
Atraso significativo	0 (0)	5 (15)	
IDADI-Cognição			0,63
Típico	2 (40)	12 (36)	
Alerta de atraso	2 (40)	8 (24)	
Atraso	1 (20)	5 (15)	
Atraso significativo	0 (0)	8 (24)	
IDADI-Socioemocional			0,19
Típico	2 (40)	18 (54)	
Alerta de atraso	3 (60)	6 (18)	
Atraso	0 (0)	6 (18)	
Atraso significativo	0 (0)	3 (9)	
IDADI-Comunicação e linguagem receptiva			0,19
Típico	4 (80)	11 (33)	
Alerta de atraso	0 (0)	7 (21)	
Atraso	1 (20)	7 (21)	
Atraso significativo	0 (0)	8 (24)	
IDADI-Comunicação e linguagem expressiva			0,56
Típico	0 (0)	1 (3)	
Alerta de atraso	0 (0)	5 (15)	
Atraso	0 (0)	4 (12)	
Atraso significativo	5 (100)	23 (69)	
IDADI – Motricidade ampla			0,61
Típico	5 (100)	24 (72)	
Alerta de atraso	0 (0)	4 (12)	
Atraso	0 (0)	3 (9)	
Atraso significativo	0 (0)	2 (6)	
IDADI – Comportamento adaptativo			0,91
Típico	3 (60)	19 (57)	
Alerta de atraso	1 (20)	6 (18)	
Atraso	1 (20)	5 (15)	
Atraso significativo	0 (0)	3 (9)	

**Legenda:** IDADI = Inventário dimensional de avaliação do desenvolvimento infantil; FE =faixa etária

## DISCUSSÃO

A análise dos resultados permite destacar a importância da avaliação do brincar, entre as crianças com TDL. Sabe-se da importância da avaliação das dimensões de comunicação e linguagem para o planejamento terapêutico, mas o brincar, enquanto espaço de troca com as crianças na sessão terapêutica tem papel fundamental no fazer do fonoaudiólogo^(21,25)^.

O estudo de Graña e Ramos^(7)^ observou que a atividade lúdica do fonoaudiólogo tem efeitos em seu fazer terapêutico. Os resultados deste estudo evidenciam a importância de o fonoaudiólogo analisar o brincar das crianças que atende para que possa fazer um planejamento terapêutico abrangente que inclua o brincar como base para o simbolismo, como vários estudos já previram na clínica da infância^(1-7,21,25,26)^. Também ressalta que o brincar espontâneo é muito mais efetivo para ancorar o desenvolvimento da linguagem do que tentativas de disfarçar exercícios de fala por meio de um brincar dirigido^(7)^.

A análise da Tabela 3 destaca esse atraso de um nível ou mais no brincar de 35 das 37 crianças pesquisadas. A maior parte dessas crianças (36) apresentavam um atraso significativo na comunicação e linguagem expressiva analisada por meio do IDADI e apenas uma criança possuía atraso na compreensão. Havia, portanto, entre as crianças diagnosticadas com TDL pelas fonoaudiólogas, um atraso importante do brincar em conjunto com o atraso na linguagem, embora isso não tenha se evidenciado estatisticamente. Seria interessante em pesquisas futuras comparar um número maior de crianças em desenvolvimento de linguagem típico, sem TDL, e crianças com TDL para verificar se a associação estatística entre a linguagem e o brincar poderia emergir.

Do mesmo modo, hipotetiza-se que não houve associação estatística entre o brincar e outras dimensões do desenvolvimento, a exceção do desenvolvimento motor fino, porque não houve alterações e variações que produzissem diferenças estatísticas no grupo estudado e pudessem se evidenciar na relação com o brincar. Este resultado foi similar ao do estudo de Mendes et al.^(12)^, que também não encontrou associação entre a maturidade simbólica, avaliada de modo similar ao modo como foi avaliado o brincar nesta pesquisa, e variáveis como vocabulário e desempenho intelectual.

Já na motricidade fina, observou-se uma variação maior, que pode estar associada ao fato de que algumas crianças da amostra apresentavam dificuldades na linguagem expressiva no âmbito de habilidades fonético-fonológicas ou articulatórias. A interface entre TDL e prematuridade tardia encontrada na amostra pode explicar os atrasos motores, também mencionados em outro estudo^(18,19)^.

Ainda em relação à idade gestacional, apesar da ausência de correlação estatística, cabe também observar que a maior parte da amostra era prematura tardia, portanto, sem grande variação na amostra. Um aspecto, no entanto, a ser ressaltado é justamente os possíveis efeitos da prematuridade tardia na linguagem, pois muito se discute na literatura os efeitos da prematuridade extrema e outros fatores como baixo peso ao nascer, tempo de internação, entre outros fatores^(21)^, mas os dados descritivos desta pesquisa sugerem que as crianças prematuras tardias merecem atenção aos seu desenvolvimento e aquisição da linguagem.

A literatura apresenta a aquisição da linguagem como um processo complexo, que depende do adequado funcionamento das estruturas encefálicas, cognitivas, constituindo-se como um processo evolutivo intricado, sendo que qualquer comprometimento em um desses elementos pode exercer um impacto significativo no desenvolvimento infantil^(18,19,27)^. Dessa forma, cabe salientar que no contexto brasileiro, anualmente, 340 mil bebês nascem prematuros, o que equivale a 931 por dia ou a seis prematuros a cada 10 minutos, sendo mais de 12% dos nascimentos no país ocorrem antes de completar 37 semanas de gestação, demonstrando um índice dobrado em comparação com países europeus^(28)^. Dessa maneira, a prematuridade emerge como um fator de risco biológico associado a possíveis alterações no desenvolvimento da linguagem infantil que deve ser considerado no acompanhamento das crianças.

Em relação à escolaridade materna e renda familiar, novamente dado o fato de que a maior parte das mães possuía escolaridade igual ou superior ao ensino médio e também acima de cinco salários mínimos talvez não se tenha sentido os efeitos desses aspectos sociodemográficos na amostra, tão ressaltados em outros estudos como um^(20)^ que correlaciona a aquisição da linguagem com a escolaridade materna e que famílias com baixos rendimentos podem apresentar cerca de 50% de atraso no desenvolvimento, mesmo após realizar ajustes para escolaridade materna.

## CONCLUSÃO

Considerando os objetivos iniciais desta pesquisa, observou-se, na análise descritiva, que crianças com Transtorno do Desenvolvimento da Linguagem possuíam brincar atrasado para a faixa etária e que o melhor desenvolvimento no brincar se associa positivamente ao melhor desempenho em motricidade fina na amostra estudada.

A ausência de associação estatística com outras dimensões do desenvolvimento e variáveis como renda familiar, idade gestacional e escolaridade materna, sugere uma homogeneidade e condições mais favorecedoras destes aspectos no grupo estudado. Ainda assim, é importante considerar que há um grande número de crianças com prematuridade tardia na amostra, o que pode ter sido um gatilho para a manifestação do TDL, do atraso no brincar e sua associação com alterações de motricidade fina. Esse fato sugere que crianças com prematuridade tardia, a exemplo da prematuridade extrema, também necessitam de um acompanhamento diferenciado na puericultura.

Cabe destacar algumas limitações do estudo que se apresentaram devido à coleta ter ocorrido no período da pandemia, o que não permitiu uma avaliação mais extensa do brincar e qualitativa da linguagem. Ainda assim, foi possível, mediante experiência das pesquisadoras, obter amostra de filmagens confiáveis para a análise do brincar, tendo em vista os procedimentos previstos na coleta da Escala Labirinto.

O estudo confirma que o brincar é tema fundamental na clínica dos distúrbios da linguagem e deve continuar a ser investigado em amostras ampliadas em estudos quantitativos futuros. Também sugere que estudos qualitativos e da dimensão psíquica do brincar sejam realizados, pois a alteração do simbolismo deve ser investigada em toda sua amplitude.
